# Evaluation of food purchasing in the Brazilian School Feeding Programme: feasibility of the requirements and recommendations

**DOI:** 10.1017/S136898002300229X

**Published:** 2023-12

**Authors:** Ana Beatriz Coelho de Azevedo, Daniel Henrique Bandoni, Ana Laura Benevenuto de Amorim, Daniela Silva Canella

**Affiliations:** 1 Rio de Janeiro State University, Institute of Nutrition, Rua São Francisco Xavier, nº 524, Maracanã, Pavilhão João Lyra Filho, 12º andar, Bloco E, sala 12002, Rio de Janeiro, RJ, Brazil; 2 Federal University of São Paulo, Center of Practices and Research in Nutrition and Collective Food Services, Edifício Central – Rua Silva Jardim, nº 136, Vila Matias, Santos, SP, Brazil; 3 Santos Metropolitan University, Avenida Francisco Glicério, nº. 06/08, Encruzilhada, Santos, SP, Brazil

**Keywords:** School feeding, Public policy, Food processing, Diversity

## Abstract

**Objective:**

To analyse the purchase of food for school feeding, according to the extent and purpose of industrial processing and variety, exploring the feasibility of achieving the requirements and recommendations of the Brazilian School Feeding Programme, and the variety of unprocessed or minimally processed foods according to the purchase of ultra-processed foods.

**Design:**

Secondary data from 2016 from the Accountability Management System of the National Fund for Educational Development, concerning the food items purchased, were used to explore the feasibility of the requirements and recommendations. The foods were grouped according to the NOVA classification system. Variety was assessed by counting different types of unprocessed or minimally processed foods.

**Setting:**

Brazil.

**Participants:**

3698 Brazilian municipalities.

**Results:**

Energy share from unprocessed or minimally processed foods was 44·1 % while that of ultra-processed foods was 29·9 %. The average of unprocessed or minimally processed food types purchased annually was 33·8 items. Of the municipalities, 35·8 % were within the limit established for the expenditure of funds for the purchase of processed and ultra-processed foods, while 8·7 % followed the recommendation for variety. The proportion of ultra-processed foods did not influence the variety of food items purchased.

**Conclusions:**

The results showed the feasibility of achieving the requirements and recommendations and underscored the importance of continued efforts to promote the inclusion of unprocessed or minimally processed foods in the school feeding programme while addressing the challenges associated with expenditure limits of processed and ultra-processed foods and enhancing variety, which is strategic to promote adequate and healthy meals.

The dietary pattern in Brazil and the world has been changing not only because of urbanisation and globalisation but also because of technological developments that have taken place in recent decades^(1–4)^. Traditional meals prepared with unprocessed or minimally processed foods are being replaced with ultra-processed foods^(5–8)^. Such a dietary pattern has also been described for children and adolescents^(9–12)^. As a result of these and other behaviour and structural changes, there has been an increase in the prevalence of non-communicable diseases, overweight and obesity^(13–16)^, including in children and adolescents^(17)^.

Recognising this scenario, the Brazilian Dietary Guidelines adopt the NOVA classification system, which categorises food items according to the extent and purpose of industrial processing, recommends using a wide variety of unprocessed or minimally processed foods, predominantly of plant origin, as the basis of the diet and advocates the avoidance of ultra-processed foods^(18,19)^. The consumption of different types and large varieties of unprocessed or minimally processed foods is advisable to improve nutrient intake and diet quality and to promote healthy and sustainable diets^(18,20)^. Two of the FAO/WHO (2019) guiding principles for healthy and sustainable dietary patterns established that diets: (1) should be based on a great variety of unprocessed or minimally processed foods, balanced across food groups, while ultra-processed foods and beverages should be restricted, and (2) should include wholegrains, legumes, nuts and an abundance and variety of fruits and vegetables. Recommending varied and diverse diets is a widely used approach; however, the concepts of variety and diversity are not always clear in the literature^(20–22)^.

According to FAO, dietary diversity is a qualitative measure of food consumption that reflects the family’s access to a variety of foods and is an indicator of the adequacy of nutrients in the individuals’ diet^(23)^. Dietary diversity is also defined as the number of different foods or food groups consumed during a given reference period. Dietary variety is considered a synonym of food diversity^(24)^. As there is no consensual definition of diversity or variety, many indicators have been proposed with many format variations. A review classified indicators into three types, count-based, evenness and dissimilarity scores. Evenness scores are used to evaluate the relative share of energy among foods consumed in the dietary pattern. Diet dissimilarity scores refer to the consumption of food with distinctive characteristics or attributes. Count-based scores are the most commonly used because of their practicality and seek to quantify the number of food groups consumed in a diet during a reference period^(25)^.

The Brazilian School Feeding Programme (*Programa Nacional de Alimentação Escolar* – PNAE) is a universal nationwide policy and the oldest in the field of food and nutrition in Brazil. In 2020, it served about 40 million students, and the federal government transferred R$ 4·3 billion to municipalities and states^(26,27)^. By providing students with adequate healthy meals and undertaking food and nutrition education initiatives, the programme is intended to help improve aspects such as students’ growth, academic achievement and food and nutrition security^(28)^. The rules for the PNAE have advanced over the years and more intensely since the 2000s. A major achievement took place in 2009, after the publication of Law No. 11 947 (16 June 2009), which implemented the PNAE across all basic education schools, included food and nutrition education as a priority for the programme, strengthened the participation of the community in the social control of the programme and formalised the guarantee of school meals to students even if the transfer of funds were to be suspended. It also made mandatory that at least 30 % of the funds transferred to states and municipalities by the federal government be used to purchase food products from family farmers^(29)^. In 2013, Resolution No. 26 (17 June 2013) was published and in addition to making recommendations for specific nutrients, it began to restrict the purchase of low-nutrient foods and beverages, mostly processed and ultra-processed foods^(30)^.

In 2020, the recommendations of the PNAE were updated to Resolution No. 6 (8 May 2020) based on important references – including the Brazilian Dietary Guidelines^(18)^ – to promote adequate and healthy food intake. In line with this objective, the PNAE recommends that most foods purchased for school meals should be unprocessed or minimally processed, in great variety and predominantly of vegetable origin, the basis of the diet; limits the supply of processed and ultra-processed foods and establishes minimum parameters for the supply of different unprocessed or minimally processed foods^(18,28)^. The varied consumption of unprocessed or minimally processed foods (such as fruits, vegetables, greens, roots, tubers, grains and cereals, legumes, eggs, milk and meat), mainly of plant origin, contributes to a nutritionally balanced diet, tasty, culturally appropriate and conducive to a socially and sustainable environmentally food system^(18)^. Additionally, Resolution No. 6 (8 May 2020) recommends that at least fifty different types of unprocessed or minimally processed foods should be offered on a yearly basis^(28)^. This recommendation can be considered a count-based score. As the terms diversity and variety are frequently used as synonyms, in the present study, we adopted the term variety.

To the best of our knowledge, there are no studies that have evaluated the relative share of foods purchased through the PNAE according to the extent and purpose of industrial processing. In the context of a new normative and its implementation, using available data to assess how prepared the country is to adapt its practices could be useful to better understand the feasibility of the requirements and recommendations. In the context of a new normative and its implementation, using available data to assess how prepared the country is to adapt its practices could be useful. Therefore, the objective of the present study was to analyse the purchase of food for school feeding, according to the extent and purpose of industrial processing and variety, exploring the feasibility of achieving the requirements and recommendations of the Brazilian School Feeding Programme, and the variety of unprocessed or minimally processed foods according to the purchase of ultra-processed foods.

## Methods

### Study design and data source

This is a cross-sectional study with 2016 secondary data from the Accountability Management System (SiGPC) of the National Education Development Fund (FNDE). The FNDE is the agency responsible for managing the PNAE, establishing rules for planning, implementing, controlling, monitoring and evaluating the programme, and transferring funds to municipalities and states. Data from the SiGPC refer to accountability carried out by all municipalities and states that receive federal funds for running the PNAE at the respective municipal and state levels. Annual accountability of all funds received is mandatory, and the SiGPC is used for this purpose^(28)^. This study addresses the feasibility of implementing the requirements and recommendations of Resolution No. 6 (8 May 2020) in Brazilian municipalities. The study included all municipalities with accountability data available in SiGPC for the year 2016 at the moment of the study.

### Database design

Data available in the SiGPC include all foods purchased by the municipalities in the respective year, as well as data on the quantity of food items purchased and the value paid (in Brazilian reais – R$). For each municipality with available data, the amount of each food item, which had been recorded in different measurement units (e.g. kg, unit, can, pack), was converted into grams. For this purpose, the table designed by Pinheiro *et al.* ^(31)^ was used to evaluate food in common household measures, and for foods not available in this table, Embrapa’s and supermarkets’ websites were used as sources of information^(32)^. Subsequently, using correction factors, the inedible fraction of foods was excluded (e.g. peels)^(33)^. The food items were then coded, and the codes were used to link the foods to the composition tables. The energy composition of the purchased foods was calculated based on the Brazilian Food Composition Table (TACO)^(34)^ and, for foods not available in this table, data from the USDA National Nutrient Database for Standard Reference were used^(35)^.

The following variables were also included in the study: regions of the country (North, Northeast, Southeast, South and Central-West), location of municipalities (capitals and non-capitals) and Municipal Human Development Index (MHDI) of municipalities. MHDI data were collected from the Atlas of Human Development in Brazil^(36)^, and the municipalities were categorised according to the tertiles of the MHDI.

### Food classification

As proposed by the NOVA classification system, which considers the extent and purpose of industrial processing, and as adopted in the Brazilian Dietary Guidelines^(18,19)^, all food items purchased by the municipalities were grouped as follows: (1) unprocessed or minimally processed foods; (2) processed culinary ingredients; (3) processed foods and (4) ultra-processed foods^(19)^. To construct the variables, in addition to the four groups proposed by the NOVA classification, a fifth group (consisting of both processed and ultra-processed foods) was created for the purpose of alignment with the Resolution No. 6 (8 May 2020)^(28)^.

Subsequently, the percentage distributions (share) of energy (kcal) and expenditure of funds (R$) were estimated for each food group out of the total energy and funds, respectively. Considering the relative share of energy from ultra-processed foods out of total energy acquired, the municipalities were categorised into quartiles of ultra-processed foods, with the first quartile (Q1) being the one with the lowest share of this group. Additionally, considering that Resolution No. 6 establishes that, at most, 20 % of the funds should be used to purchase processed and ultra-processed foods, the proportion of municipalities aligned with this limit was estimated^(28)^.

### Assessing the variety of unprocessed or minimally processed foods

Food variety was assessed by counting the different types of unprocessed or minimally processed food items purchased by each municipality. This count was used to construct the following continuous variable: number of unprocessed or minimally processed food items purchased. Additionally, considering Resolution No. 6 (8 May 2020), which recommends the annual offer of at least fifty different types of unprocessed or minimally processed foods, the percentage of municipalities that reached this recommendation was estimated^(28)^.

### Data analysis

First, a description was made of the municipalities that make up the study sample and the distribution of the total number of Brazilian municipalities, according to region, location of the municipality and MHDI. The relative share (%) of energy acquired and funds used for each food group (unprocessed or minimally processed foods, processed culinary ingredients, processed foods, ultra-processed foods and processed and ultra-processed foods) were estimated for Brazil as a whole, for the regions of the country, according to municipality location, MHDI and for each quartile of the relative share of energy from ultra-processed foods. In addition, the mean number and the median, minimum and maximum values of different types of unprocessed or minimally processed foods purchased were estimated for Brazil as a whole, for the regions of the country, according to location, MHDI and for each quartile of ultra-processed foods. The percentage of municipalities that reached the limit of funds allocated to the purchase of processed and ultra-processed foods and the percentage of municipalities that aligned with the recommendation for variety were also estimated^(28)^. Confidence intervals of 95 % (95 % CI) were calculated. The absence of overlap between intervals was assumed to be a significant mean difference, considering a significance level of 5 %. Stata software version 14.2 (StataCorp., LP) was used for all analyses.

## Results

The present study evaluated data from 3698 municipalities, equivalent to 66·4 % of the total number of Brazilian municipalities. The sample included municipalities from all regions of the country and all but one state. The distribution of all Brazilian municipalities was presented to allow a comparison with the study sample. For the MHDI, there was no difference between the means found in each of the tertiles for the study sample in comparison to the means of all municipalities in Brazil (Table 1).

**Table 1:** Distribution of the evaluated municipalities and all Brazilian municipalities. Brazil, 2016

Variables	Evaluated municipalities		Brazilian municipalities	
	*n*	%	*n*	%
Total	3698	100·0	5570	100·0
Regions				
North	298	8·1	450	8·1
Northeast	1144	30·9	1794	32·2
Southeast	1612	43·6	1668	29·9
South	195	5·3	1191	21·4
Central-West	449	12·1	467	8·4
Location				
Capitals	20	0·5	27	0·5
Non-capitals	3678	99·5	5543	99·5
	Mean	sd	Mean	sd
MHDI				
T1	0·58	0·03	0·58	0·03
T2	0·66	0·02	0·66	0·02
T3	0·74	0·03	0·74	0·03

MHDI, Municipal Human Development Index.

When analysing the mean share of food groups, according to the NOVA classification, out of the total energy purchased for Brazil, the largest percentage was found for unprocessed or minimally processed foods, with 44·07 % (95 % CI (43·79, 44·35)), followed by ultra-processed foods, with 29·88 % (95 % CI (29·62, 30·15)). The smallest percentages were found for processed culinary ingredients and processed foods, with 20·09 % (95 % CI (19·89, 20·29)) and 5·96 % (95 % CI (5·85, 6·06)), respectively. Processed and ultra-processed foods, when analysed together, had an energy share of 35·84 % (95 % CI (35·55, 36·12)) (Table 2).

**Table 2:** Mean share (%) of food groups according to the NOVA classification for total energy acquired and total expenditure of federal funds for the purchase of food in Brazil, in the regions of the country, according to location and according to the Municipal Human Development Index (MHDI). Brazil, 2016

Variables	Mean share (%) of the total energy from food groups										Mean share (%) of the total expenditure of federal funds from food groups									
	Unprocessed or minimally processed foods		Processed culinary ingredients		Processed foods		Ultra-processed foods		Processed foods and ultra-processed foods		Unprocessed or minimally processed foods*		Processed culinary ingredients*		Processed foods		Ultra-processed foods		Processed foods and ultra-processed foods*	
	%	95 % CI	%	95 % CI	%	95 % CI	%	95 % CI	%	95 % CI	%	95 % CI	%	95 % CI	%	95 % CI	%	95 % CI	%	95 % CI
Brazil	44·07	43·79, 44·35	20·09	19·89, 20·29	5·96	5·85, 6·06	29·88	29·62, 30·15	35·84	35·55, 36·12	69·32	68·88, 69·76	4·91	4·81, 5·02	5·03	4·85, 5·21	20·73	20·36, 21·10	25·77	25·35, 26·18
Regions																				
North	42·51	41·71, 43·30	21·46	20·78, 22·13	6·49	6·11, 6·87	29·54	28·69, 30·40	36·03	35·16, 36·91	64·80	63·43, 66·17	5·52	5·22, 5·83	8·00	7·16, 8·83	21·68	20·59, 22·78	29·68	28·33, 31·02
Northeast	41·41	41·04, 41·78	21·88	21·56, 22·20	6·23	6·04, 6·41	30·48	30·07, 30·89	36·71	36·28, 37·13	61·45	60·76, 62·13	6·12	5·93, 6·31	7·88	7·49, 8·27	24·55	23·93, 25·18	32·43	31·76, 33·11
Southeast	46·76	46·30, 47·22	18·63	18·31, 18·95	5·55	5·38, 5·72	29·06	28·64, 29·49	34·61	34·15, 35·07	75·43	74·84, 76·01	4·13	3·98, 4·28	2·91	2·75, 3·08	17·54	17·00, 18·07	20·45	19·88, 21·02
South	40·49	39·14, 41·85	18·28	17·40, 19·17	6·88	6·42, 7·34	34·35	33·04, 35·66	41·22	39·82, 42·63	67·68	65·79, 69·57	3·60	3·26, 3·93	4·56	4·04, 5·09	24·16	22·41, 25·92	28·72	26·82, 30·63
Central-West	43·81	42·98, 44·65	20·64	20·07, 21·22	5·96	5·66, 6·27	29·58	28·80, 30·35	35·54	34·74, 36·35	71·17	69·98, 72·36	4·83	4·54, 5·13	3·64	3·27, 4·02	20·35	19·24, 21·46	23·99	22·84, 25·15
Location																				
Capitals	41·78	37·73, 45·84	24·49	18·30, 30·69	6·79	5·34, 8·23	26·94	22·50, 31·38	33·73	28·67, 38·78	73·62	69·13, 78·12	5·35	2·35, 8·36	5·16	3·36, 6·96	15·86	12·96, 18·76	21·02	17·15, 24·90
Non-capitals	44·09	43·80, 44·37	20·07	19·86, 20·27	5·95	5·84, 6·06	29·90	29·63, 30·16	35·85	35·57, 36·13	69·30	68·86, 69·74	4·91	4·81, 5·01	5·03	4·85, 5·21	20·76	20·39, 21·13	25·79	25·37, 26·21
MHDI																				
T1	42·22	41·81, 42·63	21·68	21·38, 21·99	6·10	5·93, 6·27	30·00	29·59, 30·41	36·10	35·67, 36·53	62·19	61·49, 62·88	6·23	6·05, 6·42	7·63	7·24, 8·02	23·95	23·34, 24·56	31·58	30·90, 32·27
T2	45·09	44·60, 45·59	19·89	19·54, 20·24	5·77	5·57, 5·96	29·25	28·81, 29·69	35·02	34·54, 35·49	72·27	71·59, 72·96	4·94	4·77, 5·10	4·11	3·85, 4·36	18·68	18·10, 19·27	22·79	22·13, 23·45
T3	44·97	44·43, 45·51	18·64	18·26, 19·02	5·99	5·79, 6·19	30·40	29·89, 30·92	36·39	35·84, 36·95	73·67	72·94, 74·40	3·55	3·39, 3·70	3·30	3·10, 3·49	19·49	18·80, 20·17	22·78	22·06, 23·51

* Resolution No. 6 (8 May 2020), Section III, Art. 21: ‘On the application of funds within the scope of the PNAE: I – at least 75 % must be allocated to the purchase of unprocessed or minimally processed foods; II – a maximum of 20 % can be allocated to the purchase of processed and ultra-processed foods; III – a maximum of 5 % may be allocated to the purchase of processed culinary ingredients’.

The Brazilian region with the highest energy share for unprocessed or minimally processed foods was the Southeast with 46·76 % (95 % CI (46·30, 47·22)). By comparison, the South region had the highest energy share of the ultra-processed food group and the processed and ultra-processed food group, with 34·35 % (95 % CI (33·04, 35·66) and 41·22 % (95 % CI (39·82, 42·63)), respectively (Table 2).

The highest mean of energy for unprocessed or minimally processed foods was found in the first quartile of energy share of ultra-processed foods: 51·65 % (95 % CI (50·97, 52·34)) (Table 3).

**Table 3:** Mean share (%) of energy acquired and expenditure of funds for each of the NOVA food groups, according to quartiles of energy share from ultra-processed foods. Brazil, 2016

Food groups	Mean share (%) of the total energy from food groups								Mean share (%) of the total expenditure of federal funds from food groups							
	Quartiles of % energy from ultra-processed foods*								Quartiles of % energy from ultra-processed foods*							
	Q1		Q2		Q3		Q4		Q1		Q2		Q3		Q4	
	%	95 % CI	%	95 % CI	%	95 % CI	%	95 % CI	%	95 % CI	%	95 % CI	%	95 % CI	%	95 % CI
Unprocessed or minimally processed foods	51·65	50·97, 52·34	44·58	44·22, 44·93	41·97	41·65,42·30	38·08	37·68, 38·48	75·03	74·17, 75·89	70·82	70·06, 71·58	67·53	66·74, 68·31	63·90	62·98, 64·82
Processed culinary ingredients	22·53	22·02, 23·04	21·42	21·09, 21·76	19·68	19·38, 19·98	16·72	16·37, 17·07	5·14	4·90, 5·38	5·14	4·95, 5·32	5·09	4·90, 5·29	4·29	4·09, 4·48
Processed foods	6·02	5·77, 6·27	6·09	5·87, 6·30	5·92	5·72, 6·11	5·81	5·60, 6·01	4·90	4·53, 5·27	5·15	4·80, 5·49	5·39	5·00, 5·78	4·69	4·36, 5·03
Ultra-processed foods	19·80	19·43, 20·17	27·91	27·83, 28·00	32·43	32·34, 32·51	39·39	39·03, 39·75	14·93	14·29, 15·57	18·90	18·31, 19·49	21·99	21·38, 22·60	27·12	26·26, 27·98
Processed foods and ultra-processed foods	25·82	25·36, 26·28	34·00	33·77, 34·23	38·34	38·13, 38·56	45·20	44·81,45·59	19·83	19·04, 20·63	24·05	23·34, 24·75	27·38	26·63, 28·12	31·81	30·91, 32·72

* Minimum and maximum values of energy share from ultra-processed foods, according to quartile percentages: Q1: 0–25·49; Q2: 25·50–30·12; Q3: 30·13–34·90; Q4: 34·90–100·00.

Regarding the mean share of food groups out of the total expenditure of federal funds, the largest percentage was also found for unprocessed or minimally processed foods: 69·32 % (95 % CI (68·88, 69·76)), followed by ultra-processed foods (20·73 %; 95 % CI (20·36, 21·10)). Processed and ultra-processed foods, when analysed together, had a percentage of 25·77 % of the total expenditure of funds (95 % CI (25·35, 26·18)) (Table 2).

The Brazilian region with the highest total expenditure of funds for unprocessed or minimally processed foods was the Southeast with 75·43 % (95 % CI (74·84, 76·01)). The largest percentage of expenditure of funds for ultra-processed foods and processed and ultra-processed foods, together, was found in the Northeast region, with 24·55 % (95 % CI (23·93, 25·18)) and 32·43 % (95 % CI (31·76, 33·11)). The mean share of energy and total expenditure of funds for ultra-processed foods and processed and ultra-processed foods, together, were higher for municipalities that are not capitals, compared with capitals (Table 2).

The highest mean of expenditure of funds for unprocessed or minimally processed foods was found in the first quartile of energy share of ultra-processed foods: 75·03 % (95 % CI (74·17, 75·89)). The percentage of processed culinary ingredients and processed foods for the total expenditure of funds did not differ between the quartiles of ultra-processed foods. By comparison, processed and ultra-processed foods, when analysed together, had a higher mean expenditure of total funds (31·81 %; 95 % CI (30·91, 32·72)) in the upper quartile of energy from ultra-processed foods (Table 3).

The mean number of unprocessed or minimally processed foods purchased in Brazil was 33·77, ranging from 0 to 169, and the mean percentage of municipalities that followed the recommendation was 8·68 % (95 % CI (7·81, 9·63)) (Table 4).

**Table 4:** Distribution of the number of unprocessed or minimally processed foods purchased annually, percentage of municipalities aligned with the recommendation for variety and percentage of municipalities that reached the fund limit established for the purchase of processed and ultra-processed foods, in Brazil, in the regions of the country, according to location and according to the Municipal Human Development Index (MHDI). Brazil, 2016

Variables	Number of unprocessed or minimally processed foods purchased							Percentage of municipalities that reached the fund limit established for the purchase of processed and ultra-processed foods†	95 % CI
	Mean	95 % CI	Median	Minimum	Maximum	Percentage of municipalities aligned with the recommendation*	95 % CI		
Brazil	33·77	33·38, 34·16	34	0	169	8·68	7·81, 9·63	35·83	34·30, 37·39
Regions									
North	28·53	27·21, 29·84	28	1	83	5·37	3·31, 8·60	21·14	16·86, 26·17
Northeast	29·98	29·34, 30·63	29	0	68	4·37	3·33, 5·72	12·24	10·46, 14·27
Southeast	36·53	35·94, 37·11	36	2	169	11·17	9·72, 12·80	54·90	52·46, 57·32
South	37·05	35·46, 38·65	38	1	66	12·82	8·79, 18·33	27·18	21·37, 33·89
Central-West	35·56	34·38, 36·73	35	2	132	11·14	8·54, 14·40	40·98	36·51, 45·61
Location									
Capitals	60·95	41·00, 80·90	47	6	169	45·00	24·21, 67·70	50·00	28·17, 71·83
Non-capitals	33·62	33·24, 34·00	34	0	90	8·48	7·62, 9·43	35·75	34·22, 37·32
MHDI									
T1	29·33	28·74, 29·93	29	0	69	3·44	2·56, 4·60	16·55	14·59, 18·71
T2	33·91	33·31, 34·51	34	2	68	7·20	5·88, 8·79	44·76	41·99, 47·57
T3	38·14	37·40, 38·88	38	1	169	15·48	13·56, 17·62	46·68	43·90, 49·49

* Resolution No. 6 (8 May 2020), Section III, Art. 21, Sole Paragraph: ‘In addition, at least 50 (fifty) different types of unprocessed or minimally processed foods should be purchased annually by the municipalities’.

† Resolution No. 6 (8 May 2020), Section III, Art. 21 – On the allocation of funds within the scope of the PNAE: ‘a maximum of 20 % can be allocated to the purchase of processed and ultra-processed foods’.

There were no significant differences in distribution by region. The capital cities purchased a mean number of 60·95 different unprocessed or minimally processed foods and showed better alignment with the recommendation – 45·00 % (95 % CI (24·21, 67·70)) – compared with non-capital cities: 8·48 % (95 % CI (7·62, 9·43)). Regarding the MHDI, the last tertile presented, on average, 38·14 different unprocessed or minimally processed foods and also better alignment with the recommendation: 15·48 % (95 % CI (13·56, 17·62)) of municipalities followed this recommendation (Table 4).

Considering the funds used to purchase processed and ultra-processed foods, 35·83 % of the municipalities were within the established limit. The Southeast region – when compared with the others – had the highest percentage of municipalities within the limit of the legislation (54·90 %; 95 % CI (52·46, 57·32)). Regarding the MHDI, the last tertile showed better scenario, with 46·68 % of the municipalities (95 % CI (43·90, 49·49)) aligned with the legislation (Table 4).

Considering the energy share from ultra-processed foods, the median number of unprocessed or minimally processed foods purchased was 33 for the first and last quartiles and 34 for the second and third quartiles. Even though the median number of unprocessed or minimally processed foods is the same, municipalities with a lower energy share of ultra-processed foods showed less adequacy for the variety recommendation, when compared with those with a greater share of ultra-processed foods (Q1: 6·38 %; 95 % CI (4·97, 8·15) *v*. Q4: 10·28 %; 95 % CI (8·48, 12·41)) (Table 5).

**Table 5:** Distribution of the number of unprocessed or minimally processed foods purchased annually and percentage of municipalities that followed the recommendation for variety, according to quarters of energy share of ultra-processed foods. Brazil, 2016

Quartiles of energy share from ultra-processed foods*	Number of unprocessed or minimally processed foods purchased					Percentage of municipalities aligned with the recommendation†	95 % CI
	Mean	95 % CI	Median	Minimum	Maximum		
Q1	32·25	31·52, 32·98	33	1	74	6·38	4·97, 8·15
Q2	35·05	34·34, 35·76	34	4	90	9·52	7·79, 11·60
Q3	34·29	33·57, 35·01	34	5	80	8·54	6·90, 10·53
Q4	33·48	32·54, 34·43	33	0	169	10·28	8·48, 12·41

* Minimum and maximum values of energy share from ultra-processed foods, according to quartile percentages: Q1: 0–25·49; Q2: 25·50–30·12; Q3: 30·13–34·90; Q4: 34·90–100·00.

† Resolution No. 6 (8 May 2020), Section III, Art. 21, Sole Paragraph: ‘In addition, at least 50 (fifty) different types of unprocessed or minimally processed foods should be purchased annually by the municipalities’.

## Discussion

Using nationwide data related to the PNAE’s accountability, the present study assessed the food purchased by PNAE according to the extent and purpose of industrial processing, the variety of unprocessed or minimally processed foods and analyse the percentage of municipalities that reached the recommendation for variety and with the fund limit requirement established for the purchase of processed and ultra-processed foods. The findings showed that the largest percentage of energy acquired in Brazilian municipalities came from unprocessed or minimally processed foods, followed by ultra-processed foods. The same scenario was found for the percentage of federal funds’ total expenditure. However, the mean percentage of processed and ultra-processed foods analysed together with the total expenditure of funds was higher than the percentage provided in Resolution No. 6 (8 May 2020)^(28)^.

Resolution No. 6 (8 May 2020) establishes that at least 75 % of federal resources are allocated for the purchase of unprocessed or minimally processed foods^(28)^. The findings of the present study show that, even before the implementation of the Resolution, 69·3 % of the funds were allocated to purchase food items from this group. The resolution also establishes that, at most, 20 % of the funds can be allocated to purchase processed and ultra-processed foods^(28)^; however, it was found that 25·8 % of the funds were used to purchase processed and ultra-processed foods and over a third of the evaluated municipalities reached this recommendation.

The municipalities in the Southeast, Brazil’s richest region, and the municipalities with the highest MHDI had higher alignment with the legislation. In addition, about 9 % of Brazilian municipalities followed the recommendation of annual purchases of different types of unprocessed or minimally processed foods. The municipalities with the highest MHDI had the best scenarios, while the municipalities that used funds from the federal government to purchase a small number of foods ultimately purchased little or no unprocessed or minimally processed foods.

Although the data used in this study were produced prior to Resolution No. 6 (in 2016), we believe that the present analyses help understand the feasibility of the Resolution, since some Brazilian municipalities had already been practising the established limits and recommendations. A possible explanation for why some municipalities achieved the established limits and recommendations is that since 2013 the Resolution No. 26 (17 June 2013) has already restricted purchase of foods and beverages with low nutritional value (such as canned foods, sausages, sweets, semi-ready or ready-made preparations, soft drinks and artificial juices), most of them ultra-processed foods, and recognised the importance of variety, respect for culture and traditions for healthy eating^(30)^. Furthermore, the Brazilian Dietary Guidelines, which recommend making unprocessed or minimally processed foods, in a wide variety and predominantly of plant origin, the basis of the diet, and avoid the consumption of ultra-processed foods^(18)^, were published in 2014 and may have already influenced the PNAE.

Higher energy share of ultra-processed foods has been consistently associated with the poorer nutritional quality of diets, including lower micronutrient intake^(37)^, for which food variety and diversity can be a proxy. Although there is little research in Brazil, to date, on the subject, some studies have reported an inverse relationship between the consumption of fruits and vegetables and ultra-processed foods^(38,39)^. A nationally representative Mexican study found a nonlinear association between energy intake from ultra-processed foods and dietary diversity^(40)^. In the present study, municipalities with lower energy share from ultra-processed foods showed less alignment with the variety recommendation than those with a greater share of ultra-processed foods. This finding reinforces the importance of Resolution No. 6 (8 May 2020), which establishes the combination between the limit of funds to be used for the purchase of processed and ultra-processed foods and the recommendation of variety. It also establishes that unprocessed or minimally processed sources of Fe and vitamin A should be offered weekly to promote an adequate supply of micronutrients^(28)^.

An evaluation of Brazilian schools, based on data from the 2015 National School Health Survey, showed that 97·8 % of public schools (government-funded schools) offered meals, and their food environment was more encouraging of healthy eating than that of private schools. One of the main reasons is likely to be the implementation of the PNAE in public schools and not in private ones^(41)^. Children and adolescents spend at least part of the day at school, and previous studies have shown that students’ adherence to school meals is related to better diet quality and adequate nutritional status^(42–44)^.

School meals are often the main source of food for socially vulnerable students. A study that evaluated the relationship between food insecurity and consumption of school meals offered by PNAE in households with children and adolescents residing in Brazilian municipalities reported that most of the sampled population (56·5 %) had some degree of food insecurity, 78·5 % of students reported regular intake of school meals (>3 times/week) and students from households with moderate or severe food insecurity were more likely to regularly eat school meals^(45)^. This scenario reinforces that school meal menus should have good nutritional quality, in line with the recommendations of the Brazilian Dietary Guidelines^(18)^.

According to estimates from the World Food Programme, 388 million children receive school meals worldwide. The school meal programmes with the greatest coverage are found in India (90 million children), Brazil and China (both with 40 million), the USA (30 million) and Egypt (11 million). Together, Brazil, Russia, India, China and South Africa account for 48 % of all children who receive school meals in the world, that is, almost half of the children who receive school meals worldwide live in one of these five countries (188 million). School feeding is the most widespread social safety net worldwide in terms of the number of countries that implement this type of programme. However, school feeding coverage is lower in low-income countries and higher in high-income countries^(46)^.

The findings of the present study reinforce the importance of recommendations that restrict the supply of processed and ultra-processed foods and promote a variety of unprocessed or minimally processed foods for adequate and healthy school meals. However, unlike the PNAE, several of the largest school meal programmes in the world do not have specific recommendations about the foods they are supposed to offer. In India, intending to address hunger and education, two of the urgent issues facing most children in the country, the Mid-Day Meal Scheme provides primary and upper primary school students with a cooked meal daily. These meals are supposed to respectively contain 450 kcal and 12 g of protein and 700 kcal and 20 g of protein. China, on the other hand, runs a Nutrition Improvement Programme for students living in rural areas of the country. The programme aims to address malnutrition, improve health conditions and accelerate the development of rural education. It provides rural students with 40 % of their daily micronutrient needs. In South Africa, the National School Nutrition Programme aims to increase learning capacity and improve access to education and provide a cooked meal that is a source of protein, starch and vegetables for students in primary and secondary schools in all nine provinces in the most disadvantaged areas^(46–49)^.

PNAE is one of the oldest school meal programmes in the world and its greatest strength lies in its legal and institutional guarantees because the programme is guaranteed and regulated by a federal law that establishes the provision of school meals to all Brazilian students throughout the year. In addition, its regulations include food and nutrition education in the school curriculum, advocate the achievement of daily nutritional needs and the purchase of food from family farmers, restrict the purchase and supply of processed and ultra-processed foods, determine that school meals should be based on unprocessed or minimally processed foods and recommend the promotion of a variety of such foods^(28,46)^. Advances in PNAE’s rules can be an example for other countries and programmes because students are supposed to be provided with nutritionally adequate and healthy food.

The results found in the present study and the literature reinforce the relevance of strategies that focus not only on discouraging the consumption of ultra-processed foods but also on providing a variety of unprocessed or minimally processed foods in the school environment. Restricting the purchase of ultra-processed foods is essential to provide adequate and healthy meals at schools, but the findings of the present study reinforce the idea that restriction alone is not enough to guarantee a variety of unprocessed or minimally processed foods. Therefore, a specific recommendation is required to promote the consumption of such types of foods.

Importantly, the data collected from the SiGPC refer to the year 2016, as they were the most recent data made available by the FNDE. However, it is noteworthy that from 2013 to 2019, the programme was governed by Resolution No. 26 (17 June 2013)^(30)^. Therefore, the use of these data offers an overview of how prepared the country was to adapt to Resolution No. 6 (8 May 2020)^(28)^. In addition, they enabled to advance the analyses performed previously in the study by Canella *et al.* ^(50)^ and explore different stratifications of municipalities.

Moreover, it should be noted that the data from SiGPC do not consider the funds of the municipalities and states applied in the PNAE; they only refer to purchases made with federal funds, which are complementary. Such a situation may lead to underestimation or overestimation of the resulting percentages. However, it is believed that the use of resources should not vary according to the source of funds (federal, state or municipal governments). In addition, there were no data available from one Brazilian state and from some municipalities, which affects the representativeness of the data. The sample does not present the same distribution of municipalities by region observed for all municipalities in Brazil, since there is a greater proportion of municipalities in the Southeast and a lower participation of municipalities in the South. However, considering that the South and the Southeast are the highest income regions in the country and the combined proportion of municipalities is similar in the sample and in Brazil (48·9 % and 51·3 %, respectively), the results could not be distorted. Also, when municipalities input information, they sometimes only provide information on household measurements, that is, there was no information on weight. To compensate for such information, standardised measures were used; however, the accuracy of the values cannot be ensured.

As a strength of our study, we highlight that, to the best of the authors’ knowledge, this is the first nationwide study to analyse the purchase of food by the PNAE according to the extent and purpose of industrial processing. Another innovative aspect is that variety and diversity, assessed in the present study, are not being explored in the context of school policies and studies in this context. The findings offer an overview of how prepared the country was to adapt to Resolution No. 6 (8 May 2020)^(28)^ and reinforce the importance of providing a specific recommendation for variety.

## Conclusions

Based on nationwide data on food acquisition by Brazilian School Feeding Programme in 2016, the foods purchased for school meals in Brazil according to the extent and purpose of industrial processing and the variety of unprocessed or minimally processed foods were assessed. It was found that the largest energy share and expenditure of federal funds in Brazil came from unprocessed or minimally processed foods; however, the second largest share came from ultra-processed foods. The percentage of ultra-processed foods did not influence the variety of unprocessed or minimally processed foods.

The study also provides an overview of how prepared the country was to adapt to the programme’s new regulation. Given the findings, and following the implementation of Resolution No. 6 (8 May 2020), future assessments are expected to report a higher quality of school meals. The results can also provide further insights for assessing public policies aimed at improving the health of schoolchildren, conducting future studies on the subject and improving the performance of managers and nutritionists of the programme. Implementation studies and assessment of the impact of the policy are needed to evaluate factors that could influence compliance with these regulations at the municipality level.
